# Burnout and Quality of Life Among Massage Therapists with Visual Impairment

**DOI:** 10.1007/s10926-018-9793-7

**Published:** 2018-07-02

**Authors:** Magdalena Wrzesińska, Katarzyna Binder, Klaudia Tabała, Anna Lipert, Elżbieta Miller

**Affiliations:** 1 Department of Psychosocial Rehabilitation, Medical University of Lodz, Pl. Hallera 1, 90-647 Lodz, Poland; 2 Department of Sports Medicine, Medical University of Lodz, Lodz, Poland; 3 Department of Physical Medicine, Medical University of Lodz, Lodz, Poland

**Keywords:** Burnout, Quality of life, Physical therapists, Visual impairment

## Abstract

*Purpose* The aim of this study was to evaluate the level of burnout syndrome and quality of life (QoL) among Polish massage therapists, and determine their relationship with sociodemographic and work-related variables. *Methods* A group of 43 participants aged 28–63, who were blind or poor-sighted were recruited for the study. They were surveyed with sociodemographic data questionnaire and the Polish versions of the Maslach Burnout Inventory-General Survey and WHOQOL-BREF. *Results* The overall level of exhaustion was 6.79 ± 4.45, cynicism was estimated at 7.30 ± 3.43, and professional efficacy was 23.3 ± 5.44. Regarding QoL, the psychological domain was the highest (73.6 ± 10.0), while the physical domain was the lowest (61.1 ± 6.94). None of the sociodemographic variables or occupational factors had any statistical relationship with any burnout subscale. Significant correlations were found between the psychological domain of QoL and marital status (H = 6.570; p = 0.037), years of practice (ρ = 0.315; t = 2.124; p = 0.039), hours of practice per week (ρ = 0.364; t = 2.505; p = 0.016) and private practice (z = 2.393; p = 0.017). Significant relationships were found between the environmental domain of QoL and the place of residence (H = 5.977; p = 0.050) and between hours of practice per week (ρ = 0.335; t = 2.276; p = 0.028). A significant positive correlation was noted between professional efficacy and the social relationship domain (ρ = 0.306; t = 2.056; p = 0.046). *Conclusion* Job activity plays a crucial function in the psychosocial rehabilitation of massage therapists with visual impairment. This was confirmed by the low risk of burnout, and the psychological domain being the highest of QoL.

## Introduction

Burnout was first defined by Freudenberger in 1974 as the decrease in the power and energy of individuals who are faced with a workload [1], and 40 years later it remains a problem for human services and an urgent problem of public health [2]. Recently, the concept of burnout has been developed into a more universal form by Maslach and Leiter [3]. Nowadays, this syndrome is associated with three subscales: exhaustion (EX), cynicism (CY), and professional efficacy (PE). EX is described as a feeling of not being able to offer any more of oneself at work, while CY refers to the cynical attitudes and distance from work in general, and not only towards the beneficiaries of the services. Both subscales are the result of a dysfunctional way of coping with stress and with excessive workloads in an unsuitable environment. Finally, PE concerns feelings of decreased professional efficiency in social and non-social environments [3, 4].

Burnout is known to be one of the most important reasons for absenteeism or turnover [5], and has influenced relations between staff, co-workers and family [6]. Most studies have confirmed that age, sex, marital status, some personality traits, and a low level of job satisfaction, together with some other demographic variables, are also related to burnout [7]. Healthcare workers are at a higher risk of experiencing a number of job stressors and are more prone to forms of emotional distress such as burnout, anxiety and depression than other professionals [8].

A poor level of quality of life (QoL) could reduce work performance and it may constitute a source of burnout and early retirement among health practitioners [9]. Multiple factors related to the occupational environment such as workload, low salaries and poor work environment influence the QoL of this group [10, 11]. The World Health Organization (WHO) defines QoL as “individuals’ perceptions of their position in life in the context of the culture and value systems in which they live and in relation to their goals, expectations, standards and concerns” [12]. QoL among people with disabilities is often compared to the subjective happiness formed by a combination of one’s life satisfaction, self-image, health, functioning and socio-economic factors [13] and is determined by the same variables, regardless of ability. However, it was confirmed that despite being capable of developing and fulfilling their personal needs connected with education, job and interests, those with impairments often have less possibility to gain the same level of QoL as those without. Moreover, the QoL of people with disabilities is additionally determined by their own attitudes and by social stereotypes [14].

It should be noted that the job professional activity is seen as an important determinant of QoL [15]. The possibility to perform work is of great value, as it serves not only as a source of earnings, but also of personal development and social relationships [16].

Visual impairment (VI) is acknowledged as a major health issue. According to last available global estimates 285 million people in general population had problems with sight and 39 million of them were blind [17]. VI has a significant negative impact on the emotional, physical and social life domains, as well as work-related aspects [18, 19]. A Polish report on the QoL of people with VI found that about 60% of respondents were satisfied with their lives and a stable job was recognized as an indicator of happiness by more than half [20]. People with VI demonstrated poorer mental well-being and QoL than fully-sighted people of the same age [21], and loss of vision is more likely to affect QoL than other chronic conditions such as type II diabetes, coronary diseases or hearing impairment [19, 21]. Factors that could reduce the QoL among people with such disabilities include difficulties with adapting to everyday life, lack of independence or unpleasant emotions and experiences connected with treatment, rehabilitation and pain. Socio-economic conditions such as social marginalization, difficulties in finding a job or low material status can result in feelings of unworthiness and hopelessness [15, 22].

Many of the job categories for blind and partially-sighted people have been determined by The European Blind Union, and in the healthcare category, these include physical therapists, physiotherapists or massage therapists. Educating people with VI as massage therapists has a long tradition. In Japan, it began in the seventeenth century and has since continued as a form of rehabilitation and retraining [23]. Working as a massage therapist allows people with VI to use their residual abilities, especially tactile sense, and integrate with society by being actively employed [24].

A small number of reports indicate that burnout in massage therapists with VI is low and it has been suggested that an understanding of mental health can allow prevention of burnout in this group [23, 24]. Few studies examine the QoL among massage practitioners, especially those who are actively employed. However, one such study showed that the QoL is influenced by the work-related musculoskeletal disorders to which physical therapists are exposed, as well as working venues, workplace stress and age [25]. Therefore, the aim of the present study is threefold. It attempts to identify the level of burnout and the QoL among people with VI who are occupationally active and work as massage therapists, to identify any correlations between the level of the components of burnout and the levels of the QoL, and to identify selected sociodemographic variables and occupational factors connected with the components of burnout and QoL.

## Methods

### The Study Group

The study group comprised 43 people (13 female and 30 male) aged 28–63 (mean = 46.3 ± 8.28). All participants were active massage therapists working in health units in Poland who were blind [N = 18;(41.9%)] or poor-sighted [N = 25;(58.1%)]. Most participants had been visually impaired for more than 5 years [N = 25;(58.1%)]. All participants had been diagnosed with VI according to the International Classification of Diseases (ICD)-10 [26].

The study sample was recruited from the National Association for Blind and Partially-Sighted Massage Therapists and Physiotherapists in Poland, which is one of the sections managed by the Polish Blind Union (PBU). Nowadays, the National Association includes 50 active members. Membership requires confirmation of VI, a background in medical educational and documented membership in PBU.

All members of the association were invited to participate in the study. Interviews were conducted by one trained interviewer. The questionnaires were completed by participants via telephone and the oral personal agreement was the inclusion criteria. The exclusion criteria comprised the presence of any diagnosed intellectual disability, or any physical disability other than a visual one.

The study was approved by the Bioethics Committee of the Medical University of Lodz (no RNN/311/15/KE).

### Sociodemographic Variables and Working Variables

The sociodemographic questionnaire was constructed for the needs of this study. Information on age, gender, level of VI (blind/poor-sighted), and the age of VI onset were recorded, as well as level of education, place of residence, marital status, household structure, and whether the respondent’s incomes were the main source of livelihood. Additionally, participants were asked about the working variables such as duration of practice, hours of practice per week and if they had a private practice (Table 1).

**Table 1:** Sociodemographic variables and organizational factors of the sample

	Total	Males	Females
Age (years)			
Mean, SD	46.3 ± 8.28	46.1 ± 9.32	46.8 ± 5.96
Min	20	28	36
Max	55	63	55
Level of VI			
Blind	18(41.9)	10(33.3)	8(61.5)
Poor-sighted	25(58.1)	20(66.7)	5(38.5)
No of participants with VI			
Before 5 years	18(41.9)	10(33.3)	8(61.5)
After 5 years	25(58.1)	20(66.7)	5(38.5)
The age of VI onset			
Mean, SD	37.1 ± 11.8	36.1 ± 12.4	39.5 ± 10.5
Min	15	15	16
Max	63	63	53
Level of education			
Master of science	10 (23.2)	5 (16.7)	5 (38.5)
Technician/bachelor	33 (76.8)	25 (83.3)	8 (61.5)
Place of residence			
Residence with < 50K population	22 (51.2)	16 (53.3)	6 (46.2)
Residence with 50–500K population	12 (27.9)	6 (20.0)	6 (46.2)
Residence with ≥ 500K	9 (20.9)	8 (26.7)	1 (7.6)
Marital status			
Single	15 (34.9)	12 (40.0)	3 (23.1)
Married	24 (55.8)	16 (53.3)	8 (61.5)
Divorced/separated	4 (9.3)	2 (6.7)	2 (15.4)
Household structure			
Single person	10 (24.4)	8 (28.6)	2 (15.4)
Living with 1 person	8 (19.5)	4 (14.3)	4 (20.8)
Living with 2 or more person	23 (56.1)	16 (57.1)	7 (53.8)
The main source of incomes			
Yes	26 (60.5)	21 (70.0)	5 (38.5)
No	17 (39.5)	9 (30.0)	8 (61.5)^1^
Years of practise			
Mean, SD	18.7 ± 10.1	18.7 ± 10.3	18.6 ± 9.90
Min	0	2	0
Max	40	40	32
The age of practice onset			
Mean, SD	27.6 ± 8.45	27.4 ± 7.52	28.2 ± 10.6
Min	20	20	21
Max	55	53	55
Hours of practice/week			
Mean, SD	34.7 ± 8.06	35.4 ± 9.19	33.2 ± 4.46
Min	6	6	25
Max	50	50	40
Private activity			
Yes	22 (51.2)	19 (63.3)	3 (23.1)
No	21 (48.8)	11 (36.7)	10 (76.9)^2^

Males versus females: ^1^chi^2^=3.77; p = 0.052; ^2^chi^2^=5.88; p = 0.015

### The Quality of Life Measurement

The WHOQOL-BREF is the one of the best known instruments that has been developed for cross-cultural comparisons of QoL. It has been adopted to many languages, and its simplicity of use was the main reason for its selection in the study [27]. The Polish version of the WHOQOL-BREF was used. The questionnaire has been validated in a Polish population [28] and it enables reliable QoL measurement in healthcare staff [29]. In the present study, the Cronbach alpha reliability for the WHOQOL-BREF scale was 0.69, which indicated acceptable reliability and internal consistency in the study group. The questionnaire contained a total of 26 questions addressing two separate areas: Overall Quality of Life and General Health. A further 24 items are divided into four domains: seven items for physical health (DOM1), six items for psychological health (DOM2), three items for social relationships (DOM3) and eight items for environmental health (DOM4).

In the WHOQOL-BREF, the physical domain is connected with activities of daily living, dependence on medicinal substances and medical aids, energy and fatigue, mobility, pain and discomfort, sleep and rest and work capacity. The psychological domain consists of assessment of body image and appearance, negative feelings, positive feelings, self-esteem, spirituality/religion/personal beliefs, thinking, learning, memory and concentration. The social relationship domain incorporates personal relationships, social support and sexual activity. The environmental domain corresponds to financial resources, freedom, physical safety and security, health and social care: accessibility and quality, home environment, opportunities for acquiring new information and skills, participation in and opportunities for recreation/leisure activities, physical environment (pollution/noise/traffic/climate) and transport [27].

Each item of the questionnaire was scored on a response scale from 1 to 5. To estimate the QoL value, all raw domain scores obtained by WHOQOL-BREF were transformed to a score ranging from 4 to 20, and then transformed linearly to a 0–100 scale. All domain scores were scaled in a positive direction, with higher scores denoting higher QoL [27].

### Burnout Measurement

The Maslach Burnout Inventory-General Survey (MBI-GS) was used. It consists of 16 items that are scored on Likert scale from 0 (never) to 6 (every day) according to the frequency of occurrence of each symptom. Questions apply to the feelings and attitudes of the respondent. Burnout is understood here as “a crisis in one’s relationships with work”; this does not necessarily mean a crisis in relationships with people but is connected to cynicism about the value of personal work and doubts regarding one’s capability to perform. In contrast, engagement indicates excellent performance of work and self-confidence about effectiveness. In MBI-GS, burnout is measured on the three subscales: EX, CY and PE. The EX was taken to mean fatigue, but not necessarily caused by service recipients. CY is an expression of indifference and distant attitude towards work. PE is connected with the social and non-social aspects of occupational accomplishment, as well as worker’s expectations [30].

Higher scores on the EX and CY subscales and a lower level on the PE subscale indicate a higher level of burnout [30, 31]. The overall results were scored as the sum of items included in each scale. The mean results were measured by dividing the overall results by the quantity of items in each (for EX—5 items; CY—5 items and PE—6 items). Although the MBI-GS does not have the clinical cut-off for confirming the levels of burnout, the cut-off values were chosen based on the results of the previous study, including the following assumption: for EX, the cut-off value was ≥ 4.0 points, for CY, the cut-off value was ≥ 2.60 points, and for PE, the cut-off value was ≤ 1.50 points [30, 31]. The psychometric variables of the questionnaires have been confirmed by validation in a Polish population [4]. In our study, the MBI-GS Cronbach alpha reliability coefficient subscales were observed to vary between 0.0 and 0.69. When the MBI PE subscale was deleted, Cronbach’s alpha value was 0.69. For the CY subscale, whose reliability was 0.37, Cronbach’s alpha with MG13 deleted is 0.91: this satisfies the criterion of 0.70 for measurement instruments that have already been developed. For the EX subscale, the elimination of MG7 and MG13 improved its reliability from 0.14 to 0.87. The internal consistency for the EX subscale was 0.78, which indicated acceptable reliability and internal consistency.

### Statistical Analysis

The nonparametric Mann–Whitney U-test was used to compare the mean values of two groups of variables and independent gender groups, and the nonparametric Kruskal–Wallis test was used to compare three groups of variables. The relationships between quantitative variables were analysed using the correlation coefficient rank ρ (rho); the significance of this factor was estimated by the Students’ t test. The Chi square test was used to assess differences between subgroup and gender. The effect sizes were reported to illustrate the strength of the statistical relationships and Cohen’s d and epsilon squared (E^2^) were used. A p-value of < 0.05 was regarded as statistically significant. Statistical analysis was conducted using Statistica Software, version 10.

## Results

### Level of Burnout

**Fig. 1:**
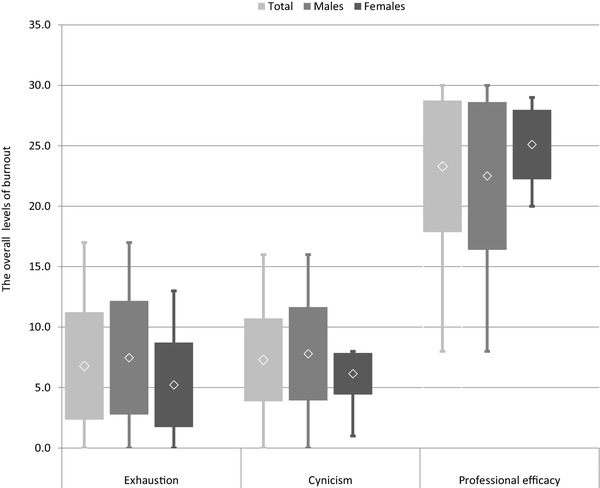
The overall levels of burnout in all subscales

The overall results were 6.79 ± 4.45 for the EX, 7.30 ± 3.43 for the CX, and 23.3 ± 5.44 for the PE. The mean levels of all subscales of burnout indicated low level burnout in the EX and CY subscales, and all participants had a low level of EX; only five participants had a high level of CY. All but one person demonstrated a low risk of burnout in the PE subscale, with only one person below the cut-off point. Although no significant differences were observed between sexes, male participants demonstrated higher overall levels of burnout in all subscales than female participants (Fig. 1).

### Level of QoL

The mean level of the Overall QoL was 4.09 ± 0.68; QoL was slightly higher among male than female respondents (4.23 ± 0.60 and 4.03 ± 0.72; z = 0.820; p = 0.412). The QoL regarding the health determinant was 3.72 ± 0.59, and this was slightly higher among women than men (3.73 ± 0.64 and 3.69 ± 0.48; z = 0.040; p = 0.968).

**Fig. 2:**
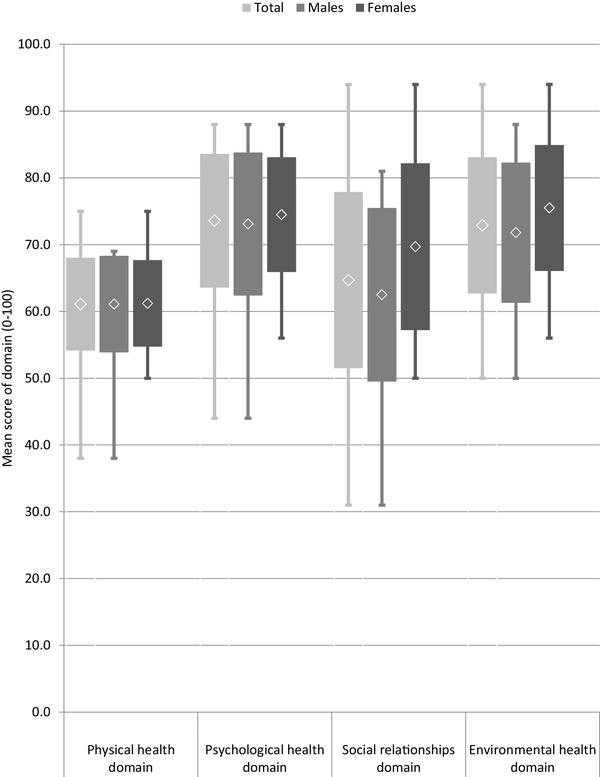
A comparison of the transformed scores (0–100-scale) of the WHOQOL-BREF in four domains according to sex

Among the whole group, the highest level of QoL was observed in the psychological domain (73.6 ± 10.0), and the lowest level in the physical domain (61.1 ± 6.94). In men, the highest QoL was found in the psychological domain (73.1 ± 10.7), while in women, it was found in the environment domain (75.5 ± 9.43). The physical domain was the lowest, both in men and women (61.1 ± 7.24 and 61.2 ± 6.48, respectively). Women reported higher scores than men in the psychological domain (74.5 ± 8.60 and 73.1 ± 10.7), sociological domain (69.7 ± 12.5 and 62.5 ± 13.0) and environmental domain (75.5 ± 9.43 and 71.8 ± 10.5). Men and women demonstrated very similar scores in the somatic domain; the differences between the sexes were not statistically significant (Fig. 2).

### Sociodemographic Variables, Working Variables and Level of Burnout and QoL

No sociodemographic variables or occupational factors had any significant relationship with any of the burnout subscales. Similarly, no significant relationship was found between overall QoL, and sociodemographic variables and occupational factors. Only place of residence had a significant relationship with health-related QoL (H = 7.467; p = 0.024; E^2^ = 0.178) (Table 2).

**Table 2:** The correlation between the level of quality of life, burnout and sociodemographic variables and occupational factors

Variables	EX	CY	PE	Overall QOL	Health QOL	Physical domain	Psychological domain	Social relationship domain	Environmental domain
Age (years)									
ρ	− 0.059	− 0.109	− 0.013	0.033	0.130	− 0.188	0.104	0.266	0.063
T	0.378	0.703	0.086	0.214	0.838	1.228	0.667	1.769	0.407
Level of VI									
Blind	6.61 ± 4.29	7.61 ± 2.70	21.4 ± 6.27	4.12 ± 0.33	3.71 ± 0.59	60.7 ± 8.34	73.9 ± 11.5	66.6 ± 12.0	73.3 ± 12.0
Poor-sighted	6.92 ± 4.65	7.08 ± 3.90	24.6 ± 4.42	4.08 ± 0.84	3.73 ± 0.60	61.4 ± 5.90	73.3 ± 9.04	63.2 ± 14.0	72.6 ± 9.05
Z	0.086	0.997	1.625	0.062	0.087	0.062	0.590	0.542	0.505
Participants with VI									
Before 5 years	6.61 ± 4.29	7.61 ± 2.70	21.4 ± 6.27	4.11 ± 0.83	3.72 ± 0.67	60.7 ± 8.34	73.9 ± 11.5	66.6 ± 12.0	73.3 ± 12.0
After 5 years	6.92 ± 4.65	7.08 ± 3.90	24.6 ± 4.42	4.08 ± 0.57	3.72 ± 0.54	61.4 ± 5.90	73.3 ± 9.04	63.2 ± 14.0	72.6 ± 9.05
Z	0.086	0.997	1.625	0.554	0.086	0.062	0.590	0.542	0.505
The age of VI onset									
ρ	0.059	0.041	− 0.197	0.073	− 0.054	− 0.078	0.170	0.107	0.080
T	0.376	0.265	1.289	0.471	0.344	0.499	1.105	0.687	0.514
Level of education									
Master of science	6.30 ± 4.16	7.20 ± 1.99	25.2 ± 4.57	4.20 ± 0.42	3.70 ± 0.67	60.9 ± 6.79	73.8 ± 11.8	68.8 ± 7.32	75.0 ± 6.32
Technician/bachelor	6.94 ± 4.58	7.33 ± 3.78	22.7 ± 5.60	4.06 ± 0.75	3.73 ± 0.57	61.2 ± 7.09	73.5 ± 9.62	63.4 ± 14.3	72.3 ± 11.2
Z	0.230	0.144	1.351	0.474	0.158	0.086	0.201	0.992	0.834
Place of residence									
Residence with < 50K	7.78 ± 4.44	6.95 ± 3.17	24.7 ± 4.72	4.18 ± 0.66	3.86 ± 0.56	61.6 ± 5.85	75.8 ± 6.49	65.0 ± 13.9	76.9 ± 8.65
Residence with 50–500K	4.92 ± 4.56	8.17 ± 3.51	21.0 ± 6.78	4.08 ± 0.51	3.33 ± 0.49	60.3 ± 9.03	70.3 ± 9.19	66.1 ± 12.0	69.8 ± 10.7
Residence with ≥ 500K	6.89 ± 3.95	7.00 ± 4.09	22.9 ± 4.46	3.89 ± 0.93	3.88 ± 0.60	61.2 ± 7.03	72.3 ± 16.4	61.8 ± 13.9	67.4 ± 10.2
H	3.842	0.630	3.014	0.457	7.467^1^	0.102	2.396	0.473	5.977^2^
Marital status									
Single	8.27 ± 5.60	8.80 ± 4.66	22.3 ± 5.14	3.80 ± 0.68	3.47 ± 0.52	61.5 ± 6.90	68.0 ± 12.0	61.6 ± 14.0	70.5 ± 10.2
Married	6.42 ± 3.62	6.79 ± 2.13	23.2 ± 5.77	4.25 ± 0.68	3.88 ± 0.61	60.9 ± 7.11	76.0 ± 7.41	65.6 ± 13.0	72.8 ± 10.1
Divorced/separated	3.50 ± 1.91	4.75 ± 2.50	27.5 ± 2.65	4.25 ± 0.50	3.75 ± 0.50	61.3 ± 8.02	79.8 ± 7.89	70.3 ± 10.7	80.3 ± 6.27
H	3.599	4.100	3.609	3.820	4.268	0.018	6.570^3^	1.331	5.052
Household structure									
Single person	8.10 ± 6.47	9.40 ± 5.19	22.8 ± 4.16	3.80 ± 0.63	3.40 ± 0.52	59.6 ± 6.29	67.0 ± 13.5	63.1 ± 10.9	69.4 ± 8.09
Living with 1 person	6.00 ± 2.27	5.75 ± 2.05	27.1 ± 2.64	4.25 ± 0.71	4.00 ± 0.53	64.3 ± 6.56	77.3 ± 10.0	68.8 ± 16.1	76.6 ± 12.5
Living with 2 or more person	6.52 ± 4.21	6.91 ± 261	22.7 ± 5.86	4.17 ± 0.72	3.74 ± 0.62	60.9 ± 7.49	75.0 ± 7.43	63.6 ± 13.3	72.4 ± 10.1
H	0.450	4.214	5.238	2.092	4.664	1.970	4.508	2.088	3.090
The main source of income									
Yes	6.92 ± 4.89	7.42 ± 3.96	23.4 ± 5.05	4.12 ± 0.71	3.73 ± 0.60	61.3 ± 6.13	72.7 ± 11.6	63.9 ± 13.4	73.0 ± 9.76
No	6.59 ± 3.81	7.12 ± 2.50	23.1 ± 6.13	4.06 ± 0.66	3.71 ± 0.59	60.9 ± 8.23	74.9 ± 7.20	65.8 ± 13.2	72.9 ± 11.0
Z	0.050	0.509	0.062	0.224	0.087	0.012	0.385	0.124	0.012
Years of practice									
ρ	− 0.037	− 0.032	0.018	0.200	0.161	0.089	0.315	0.284	0.156
T	0.234	0.205	0.114	1.305	1.042	0.572	2.124^4^	1.896	1.014
The age of practice onset									
ρ	0.028	− 0.051	0.008	− 0.268	− 0.036	− 0.199	− 0.220	− 0.274	− 0.276
T	0.177	0.328	0.052	1.778	0.233	1.301	1.441	1.827	1.841
Hours of practice/week									
ρ	− 0.051	0.034	0.034	0.190	0.148	0.211	0.364	0.191	0.335
T	0.327	0.217	0.221	1.237	0.956	1.382	2.505^5^	1.247	2.276^6^
Private activity									
Yes	7.68 ± 4.65	7.18 ± 3.84	24.3 ± 4.91	4.14 ± 0.77	3.77 ± 0.61	62.1 ± 6.24	76.7 ± 10.1	63.0 ± 12.2	73.7 ± 8.19
No	5.86 ± 4.13	7.43 ± 3.03	22.2 ± 5.87	4.05 ± 0.59	3.67 ± 0.58	60.1 ± 7.62	70.2 ± 8.99	66.4 ± 14.2	72.1 ± 12.2
Z	1.288	0.535	1.191	0.413	0.413	0.474	2.393^7^	0.814	0.085

^1^p = 0.024; ^2^p = 0.05; ^3^p = 0.037; ^4^p = 0.039; ^5^p = 0.016; ^6^p = 0.028; ^7^p = 0.017

Neither the physical domain nor the social relationship domain had any significant relationship with any sociodemographic variables or occupational factors (Table 2). A significant correlation was found between the psychological domain and marital status (H = 6.570; p = 0.037; E^2^ = 0.156). Additionally psychological domain was positively correlated with years of practice (ρ = 0.315; t = 2.124; p = 0.039), hours of practice per week (ρ = 0.364; t = 2.505; p = 0.016) and private practice (z = 2.393; p = 0.017; d = 0.679). A significant relationship was also found between the environmental domain and place of residence (H = 5.977; p = 0.050; E^2^ = 0,142) and a significant positive correlation with hours of practice per week (ρ = 0.335; t = 2.276; p = 0.028).

### Correlation Between Burnout and QoL

No significant correlation was found between the level of EX, CY and any of the domains of QoL and overall and health-related QoL. Only PE was found to have a significant positive correlation with social domain (ρ = 0.306; t = 2.056; p = 0.046) (Table 3).

**Table 3:** The correlation between burnout and quality of life level

	Exhaustion	Cynicism	Professional efficacy
Overall QoL			
ρ	− 0.152	− 0.293	0.079
t	0.987	1.960	0.507
Health QoL			
ρ	− 0.076	− 0.261	0.081
t	0.486	1.733	0.522
Somatic domain			
ρ	0.139	− 0.172	− 0.137
t	0.897	1.121	0.884
Psychological domain			
ρ	0.148	− 0.050	− 0.108
t	0.959	0.323	0.698
Social relationship domain			
ρ	− 0.177	− 0.085	0.306
t	1.151	0.549	2.056^1^
Environmental domain			
ρ	− 0.064	− 0.085	0.298
t	0.411	0.549	1.996

^1^p = 0.05

## Discussion

A low level of burnout was observed in all subscales among massage therapists. These results are consistent with a previous study which indicated a low level of burnout in this group [24]. Our findings indicate that the low level of burnout observed among massage practitioners was related with a good level of occupational environment. It is also likely that job activity has a positive impact on the psychosocial rehabilitation of people with VI.

The therapists are also more prone to burnout than other health professionals, because they are usually in close interaction with their patients during the rehabilitation process. Physical therapists are seen as a group vulnerable to burnout because of the specific nature of their relationship with chronically ill, aggressive and depressive patients [32]. The risk of burnout in this group could be increased by stressful work conditions as lack of autonomy, disorganization in the hierarchical chain of command, lack of professional and social recognition or interpersonal conflict with superiors [33]. High time pressure, work demands, heavy workload and staff shortages could also result in low disengagement from the job or job dissatisfaction in this group [34]. It could also worsen the functioning and quality of healthcare by increasing the risk of medical errors, and impairing empathy and communication with patients, which could ultimately result in a low level of patient satisfaction [35].

We assume that our massage therapists with VI were less prone to burnout than other therapists because they have more resources to allow them to work with patients with impairments. Their own success in the psychosocial rehabilitation process, possibly confirmed by their success in being actively employed, may further support and strengthen their process of rehabilitation. It could also provide them with better tools to understand the needs of patients, allowing them to be better able to stimulate patient motivation or cope with aggressive or depressive reactions.

The results from our study confirm that male participants achieved a higher level for EX, CY and PE than female participants, but the difference was not significant. There is no strong evidence that the level of burnout is dependent on sex, but some results indicate that women are more prone to burnout than men [36] which can be accounted for by differences in personal life or lower positions in the professional environment [4]. A Polish study concerning the validation of the MBI-GS questionnaire initially assumed that women present a higher EX score than men because of additional housework duties, but a lower CY score due to their greater tendency to seek fulfillment outside professional life and obtain more support from family members. In addition, men were expected to demonstrate higher PA scores, because they are typically promoted more quickly and receive higher remuneration than a woman who is employed in the same position. However, in contrast, the results reveal no significant differences between the sexes in Poland with regard to EX or PE; significant differences were only observed in CY, where women obtained a lower mean score than men [4].

Some demographic and organizational variables such as gender, being less-educated or working in a hospital have a significant influence on the development of burnout [37]. Our present findings indicate that sociodemographic and work-related variables have no significant impact on the level of burnout, which is consistent with previous results [38]. However, it should be noted that our participants who declared single marital status have previously reported a higher level of burnout than those who were married or divorced. Some reports suggest that single people were at a greater risk of burnout than married ones [39]. However, work-family conflict has recently been the source of increasing pressure in professional life; this is particularly more observed among women because of the overload, stress or conflicts associated with work-related and family-related roles [40].

Job activity has a vital meaning for QoL and unemployment has a substantial, negative effect on QoL in the general population [41]. For people with impairments, job activity not only provides economic independence and self-realisation, but it also has a therapeutic aspect in that it fosters greater self-esteem and a sense of usefulness. Job activity realises important aim of rehabilitation [15]. It was confirmed that higher QoL was found among people with disabilities who were actively employed [42]. VI affects QoL by reducing social independence and social interaction, which also influences also daily activities, mobility and social participation. A significant correlation was confirmed between QoL and employment status among people with VI. It was also reported that employment, social presence or financial independence could improve QoL in that group [43].

Our findings indicate that our participants obtained the highest level of QoL in the psychological domain, but the lowest in the somatic domain. In contrast, health-care staff reported their QoL to be highest in the physical domain, but lowest in the environmental domain [29]. It could be argued that although massage therapists are independent in their occupational activity, they are still dependent on medical care and they are aware of their fatigue regarding daily functioning. On the other hand, the fact that QoL was highest in the psychological domain in this group may reflect the positive impact of job engagement on the assessment of body image and appearance, positive thinking, cognitive processes and level of self-esteem or spiritual belief. Our results indicate that marital status, years of practice, hours of practice per year and running one’s own practice have a significant influence on the psychological domain of QoL. The autonomy and independence in their work life probably allowed them to engage fully in the development of their career.

Our study found that while women tended to demonstrate slightly lower general QoL, they also recorded slightly higher scores throughout the health-related QoL domain. Generally, women are prone to lower QoL scores than men in the occupationally active population [44]. This has been associated mainly with asymmetric life roles throughout life: men tend to experience a more continuous career, and women are regarded as playing a central role in family life. Gender differences have also been noted among people with VI [44]. However, previous studies on healthcare professionals suggest that women have lower QoL than men [29].

The main conclusion from our survey is that higher social QoL corresponds to greater personal efficacy. Previous studies suggest that social support and having family and other relationships serve a protective function against burnout [38]. Additionally, social support and autonomy are seen as resources that motivate and increase work engagement [45]. We hypothesize that these are bidirectional relations in this context. Persons with disabilities who have social support feel more effective, can develop their strengths and reduce weaknesses, gain feedback and view themselves as valued members of society; in addition, with others can change the self-image of people with disabilities from one of being a disabled person, to being an effective worker. All these are important factors that can serve as motivational drives to take part in a more active life. Those with disabilities in active employment can gain support from other resources i.e. from childhood and family, that assist them in their independence. Hence, not only support during productive age significantly improves work activity and QoL, but the earlier socializing process also plays a role. It should be noted that all respondents in this survey were members of an association, which could support both professional development and the social aspect of QoL [46]. Additionally, some of the participants work in public institutions and have families, and as such are likely to have constant contact with people without impairments. Incorporating people with impairments in different social networks allows them to focus more on taking advantage of their resources than on their deficits [16].

Our study has some limitations: the study is based on a small number of participants and the lack of similar studies makes it difficult to generalize our results. Nonetheless, our findings do indicate reinforce the need for the development of further studies confirming the positive impact of professional work on the functioning of people with VI. Moreover, the future studies should determine the prevalence and the association between work-related musculoskeletal disorders and quality of life among physical therapist with VI. It should be noted that low vision is associated with high risk of developing musculoskeletal problems, especially in the neck/scapular area [47]. Additionally, the high prevalence of musculoskeletal disorders were observed among physical therapists and this had negative impact on the quality of life and stress in the work [25].

## Conclusion

It was found that the studied massage therapists were at low risk of burnout. Of the examined domains, the psychological domain demonstrated the greatest QoL and the physical domain the least. Additionally, high QoL was found in the social domain, corresponding to higher personal efficacy. Based on this data, we conclude that job activity among people with VI plays a crucial function in psychosocial rehabilitation. To improve the social functioning of this group, we recommend the implementation of programs based on professional activation, including continuous education aimed at preparation for employment, and professional counselling at all ages. Another crucial task would be to encourage and support employers in creating jobs for people with disabilities.
